# Mapping the national HPC ecosystem and training needs: The Greek paradigm

**DOI:** 10.1007/s11227-023-05080-y

**Published:** 2023-02-15

**Authors:** Stelios Karozis, Xenia Ziouvelou, Vangelis Karkaletsis

**Affiliations:** 1 Institute of Nuclear and Radiological Sciences and Technology, Energy and Safety, National Centre for Scientific Research “Demokritos”, Patriarchou Grigoriou & Neapoleos, Agia Paraskevi, 15310 Attica, Greece; 2 Institute of Informatics and Telecommunications, National Centre for Scientific Research “Demokritos”, Patriarchou Grigoriou & Neapoleos, Agia Paraskevi, 15310 Attica, Greece

**Keywords:** HPC, Supercomputing, Training needs analysis, Education

## Abstract

HPC is a key tool for processing and analyzing the constantly growing volume of data, from 64.2 zettabytes in 2020 to an expected 180 zettabytes in 2025 (1 zettabyte is equal to 1 trillion gigabytes). As such, HPC has a large number of application areas that range from climate change, monitoring and mitigating planning to the production of safer and greener vehicles and treating COVID-19 pandemic to the advancement of knowledge in almost every scientific field and industrial domain. The current work presents an HPC Training Mapping Framework and the relevant findings and processed data of an online Training Needs Analysis (TNA) survey. The latter was used to map the training demands and gaps of existing skills and future ones. The participants consist of academia and industry and the data were utilized to find the profile of HPC user alongside the best training practices that are in need. It is found that in Greece during the year 2021, the stakeholder segment with the highest number of respondents was from academia and research with a total of 74%. The vast majority appear to have basic information accounting for 37% of the respondents. In terms of familiarity, users with intermediate familiarity with HPC represented 21% of respondents, followed by non-familiar users that accounted in total for 16.1. Advanced and highly advanced user segments account only for 8.6% and 7.4% accordingly. Overall, it is found that a: (1) fast-pace, (2) entry level, (3) applied HPC training but (4) not focused only on HPC, that will (5) provide some kind of certification, by the Greek HPC ecosystem.

## Introduction

In today’s information technology society, industries are through the fourth industrial revolution (also known as Industry 4.0). Technology, talent, and new technology enabled innovation ecosystems are emerging building greater complexity into our final innovation offerings. In this landscape the exponential growth of computational power of high-performance computing (HPC or supercomputing) is accelerating various activities for technological innovation in most enterprises [1, 2]. HPC has a strong ability of transcendental speed for calculation and data analysis. Thus, with using HPC capability, industry has the potential to accomplish discovery and technological innovation in various industry fields. As such, HPC usage and applications may have a crucial influence on future national economy [3]. The latter make the training of HPC experts important in order to accommodate the future needs and the arising demand.

In the past years, a series of actions and frameworks were implemented toward the diffusion of HPC benefits and services mainly addressing the academic community and high-skilled programmers or administrators [4–7]. As a result, the academic community is a heavy user of HPC infrastructures as the simulation of complex physical systems or the analysis of massive experimental or observational data (big-data), demand large computational power and parallelization that cannot be met by personal computers. On the other hand, integrating HPC services in business is a hard task with a small portion of very large industries investing in HPC for R&D purposes. New tools and ideas are essential to be created for introducing the benefits of HPC to industry. Lee and Jeong [8] attempt to provide a familiar way to business by utilizing the business building blocks model. Most recently, specialized MSc courses were created for introducing graduate students to HPC more massively and organized [9–11]. The latter is the natural continuation of the HPC technology to become mainstream and part of industrial R&D and production workflow. This is also promoted by the EU commission that identifies HPC as one of the key digital domains for Europe. As such, it has become a strategic investment priority [12], playing a significant role in Europe’s path toward recovery [13], with an EU investment expected to significantly increase in the next Multiannual Financial Framework (2021–2027) [14]. The goal is to promote HPC services outside academia and toward the creation of a new HPC ecosystem and potential experts that could transfer the benefits of HPC to industry and public sector.

In this concept, it is important to assess the ecosystem of HPC current and future users, their familiarity with HPC and at the same time to identify, understand and articulate the distinct training HPC needs as well as those that want to become HPC users.

In the current study, the outcome of a survey study (Training Needs Analysis (TNA)) [15] will be presented that was designed and implemented, during 2021, for the Greek HPC ecosystem. The findings and training needs mapping will help to identify demand, discrepancies, or gaps between current skills and skills required for the effective HPC implementation across various stakeholder segments with specific HPC needs. As such, they can be used as basis in order to facilitate the development of specialized training portfolios [16–18] for the coming years, aiming to enhance the skills and the use of High-Performance Computing in Greece

## Methodology

A first step to advance competitiveness in research, improve effectiveness of government services and promote innovation in industry, is to assess the Greek ecosystem of HPC current and future users, and their familiarity with HPC and at the same time to identify, understand and articulate the distinct training HPC needs of the Greek users as well as those that want to become HPC users. As such a survey study (Training Needs Analysis (TNA)) was designed and implemented, during 2021, and the findings consist of description of the Greek HPC ecosystem and training needs. The latter will identify needs, discrepancies, or gaps between current skills and skills required for the effective HPC implementation across various stakeholder segments with specific HPC needs. Moreover, they will facilitate the development of specialized training portfolios for the coming years, aiming to enhance the skills and the use of High-Performance Computing in Greece.

**Fig. 1:**
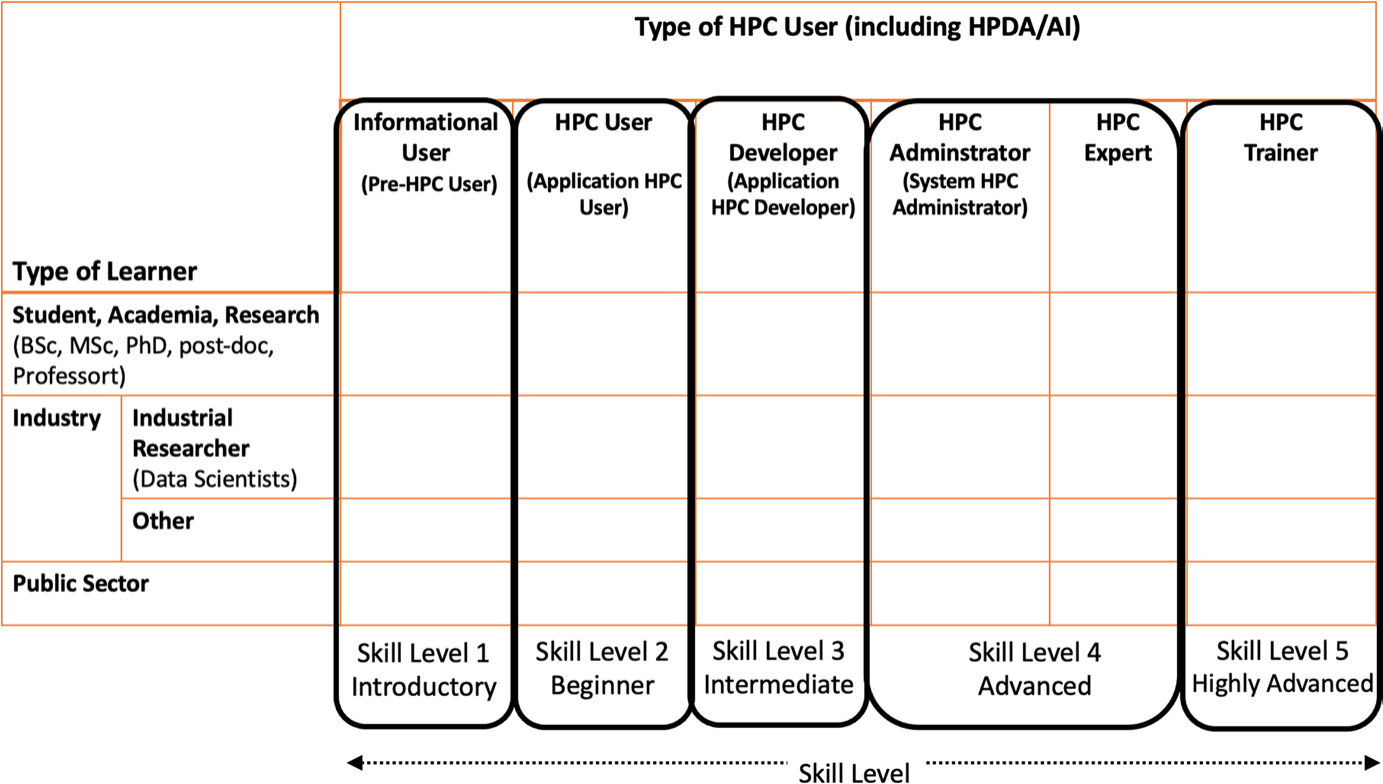
An HPC Training Mapping Framework [15]

In the current study, a training mapping framework has been developed [15] (see Fig. 1) that depicts in the horizontal axes, the different types of HPC Users and their associated skill level, into five key categories:

- Level 1: Informational (pre-HPC) type of HPC user—Introductory skill level
- Level 2: HPC User (application user)—Beginner skill level
- Level 3: HPC Developer (application developer)—Intermediate skill level
- Level 4: HPC Expert type of user and HPC Administrator type of user—Advanced skill level
- Level 5: HPC Trainer—Highly Advanced skill level

In the vertical axes, the types of learner stakeholder segments, into four categories:

- Academia/Research: including both students and the researchers (PhD and postdoctoral researchers), professors
- Industry: (a) Industrial Researcher: Data Scientists and research software engineers from industrial communities (research positions only) and (b) Other non-research industrial positions in small- and medium-sized enterprises (SMEs) and large companies
- Public Sector: including all public sector organizations

This framework has facilitated both the creation of the survey, and will also enable the depiction of the current study findings in order to understand the HPC training needs of the Greek users (“demand” side of the HPC training).

The research methodology for identifying the HPC training needs in Greece, involved an online questionnaire survey. The questionnaire design involved four thematic sections: (1) generic information (individual information and background), (2) HPC Familiarity and Usage, (3) HPC Training Needs, and (4) Suggestions. (see Supporting Information). Overall nine questions were included, using a Likert-type scale (5 or 7 type scale) and response drop-down boxes or selection of single responses by ticking boxes. Open-ended responses were included in specific questions aiming to enable participants to provide additional information and express their views.

To facilitate the dissemination of the survey, an online questionnaire [19] was created utilizing as a survey platform Google Forms, respecting all ethical and privacy considerations (informed consent, privacy and confidentiality, right to withdrawal) [20]. The questionnaire was compiled based on (1) the empirical needs of the academia and industry HPC community and (2) taking into account the HPC Training Mapping Framework. Then it was tested and refined via the EuroCC@Greece partners, before becoming publicly available. The study started in October 2021 and ended in December 2021, covering a period of three months. The sampling process involved the EuroCC@Greece [21] partners networks as well as online dissemination of the survey in order to ensure a random sample of respondents.

The survey was disseminated to all relevant stakeholders (academia, industry and public sector) aiming to identify their training needs in relation to HPC, High Performance Data Analytics (HPDA), Artificial Intelligence (AI). The participation was adequate and more than 80 replies were received.

## Results and discussion

In the next sections, the analysis of the profile, HPC usage and familiarity, as well as training needs are presented.

### User profile and skills

Based on the current study, the elements that comprise the profile of the Greek HPC user are the position the stakeholders currently have, alongside the scientific and/or expertise domain. It is found that the stakeholder segment with the highest number of respondents was from academia and research (professors, researchers, and students) with a total of 74% (see Appendix 1).

The Engineer & Tech had the highest share accounting for 40.6%, followed by Natural sciences (26%) and computer and information sciences (25.9%). Other categories are under 10% each, due to the lack of participants or to the kind of research that does not utilize HPC services, so far (see Appendix 1).

**Fig. 2:**
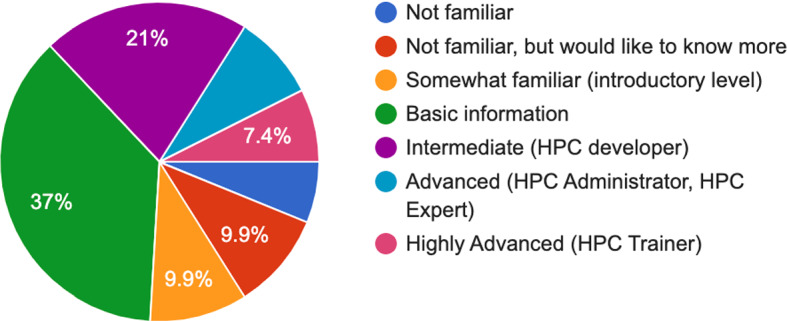
Pie chart of user familiarity with HPC [$N=81$] (color figure online)

Having identified that the profile of the survey participants comes mainly from Academia, an assessment of the respondents familiarity of HPC infrastructure and/or usage was performed. As it can be seen in Fig. 2, the vast majority appear to have basic information (Level 2: Beginner HPC user level, see the training mapping framework in Fig. 1) accounting for 37% of the respondents. Users with intermediate familiarity with HPC (Level 3: Intermediate HPC user level), represented 21% of our respondents, followed by non-familiar users that accounted in total for 16.1%, comprising users that are either not familiar (6.2%) or not familiar but interested in learning more (9.9%). Advanced (Level 4: HPC Expert, HPC Administrator) and highly advanced (Level 5: HPC Trainer) user segments account for 8.6% and 7.4% accordingly.

**Fig. 3:**
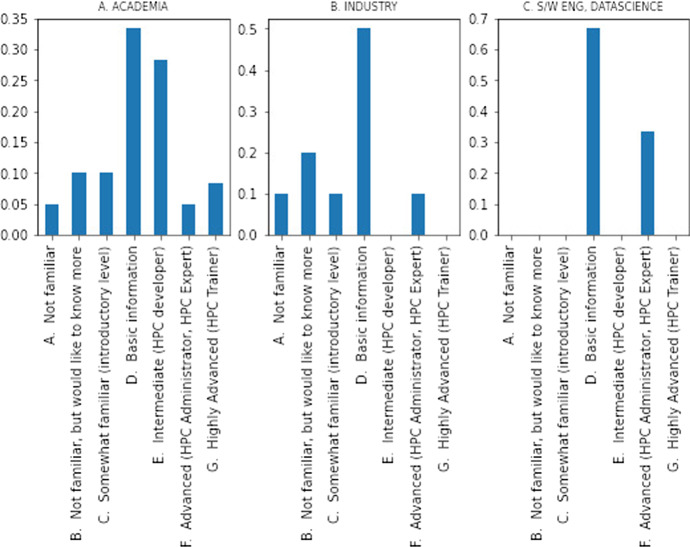
Bar plots familiarity of HPC over type of user [$N=81$]. The individual *x*-axis describes the level familiarity and *y*-axis the percentage of sample per each position

Figure 3 presents the bar plots per type of user (i.e., user’s position/occupation), with *x*-axis describing the level familiarity and *y*-axis the percentage of sample accordingly. In the case of academia/research a normal distribution of the levels of familiarity exists. Whereas, in industry (non-industrial positions) a positive skewed distribution depicts the low diffusion of HPC services in the sector. However, when the S/W engineers and Data Scientist (industrial researchers) are considered, which constitutes a category of their own, we can see that they are either advanced users or have basic information for the subject due to their background. These findings validate the classification framework, since if we considered Industry as a single category, the industrial research community would have been masked inside this category providing us with ambiguous results.

### Training needs and preferred training schemes

**Fig. 4:**
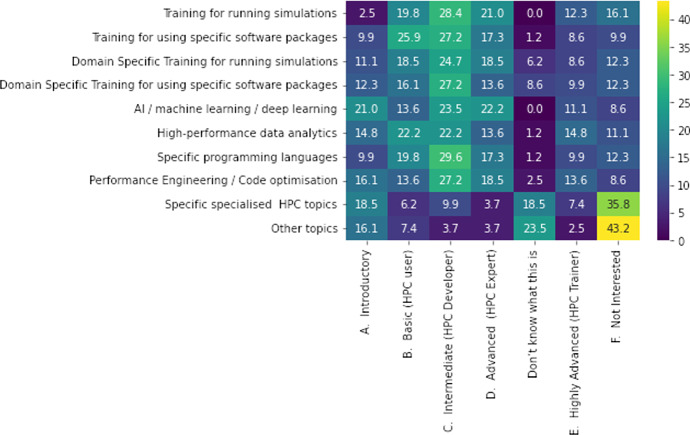
Map of training needs and level of experience [$N=81$]. The *x*-axis is the level of training difficulty and the *y*-axis the training category. The coloring goes from dark purple (0%) to yellow (100%) depending on the counts in the sample (color figure online)

Aligned with the dual scope of this survey study, from the one side (1) to assess the profile, field and familiarity of the users in Greece and from the other side (2) to estimate the training needs of the academia, industry and miscellaneous ecosystem, we now focus on the latte. A series of training categories were offered to the participants of the questionnaire to choose. The results are presented in Fig. 4 as a heatmap plot.

At first glance, the “intermediate” level training (Level 3: HPC developer, Application Developer) accounted for a quarter of the answers (25%) for all the categories. The only exception is “Specific, specialized HPC topics”, and “Other topics” categories, that present high percentages of “not interested” answers. In the same categories, a small percentage of participants appear to be interested in introductory training. As data becomes a high value commodity, data analytics and AI are topics that are becoming more well-known and more people have started exploring the possibility to integrate them into their production (academia or industry). The latter is captured in the current survey. There is high demand for introductory and basic AI/machine learning/deep learning and High Performance Data Analytics (HPDA) training. Overall, it is found that HPC users, or potential users, demand low to intermediate level of training, with advanced and highly advanced not fitting their needs.

**Fig. 5:**
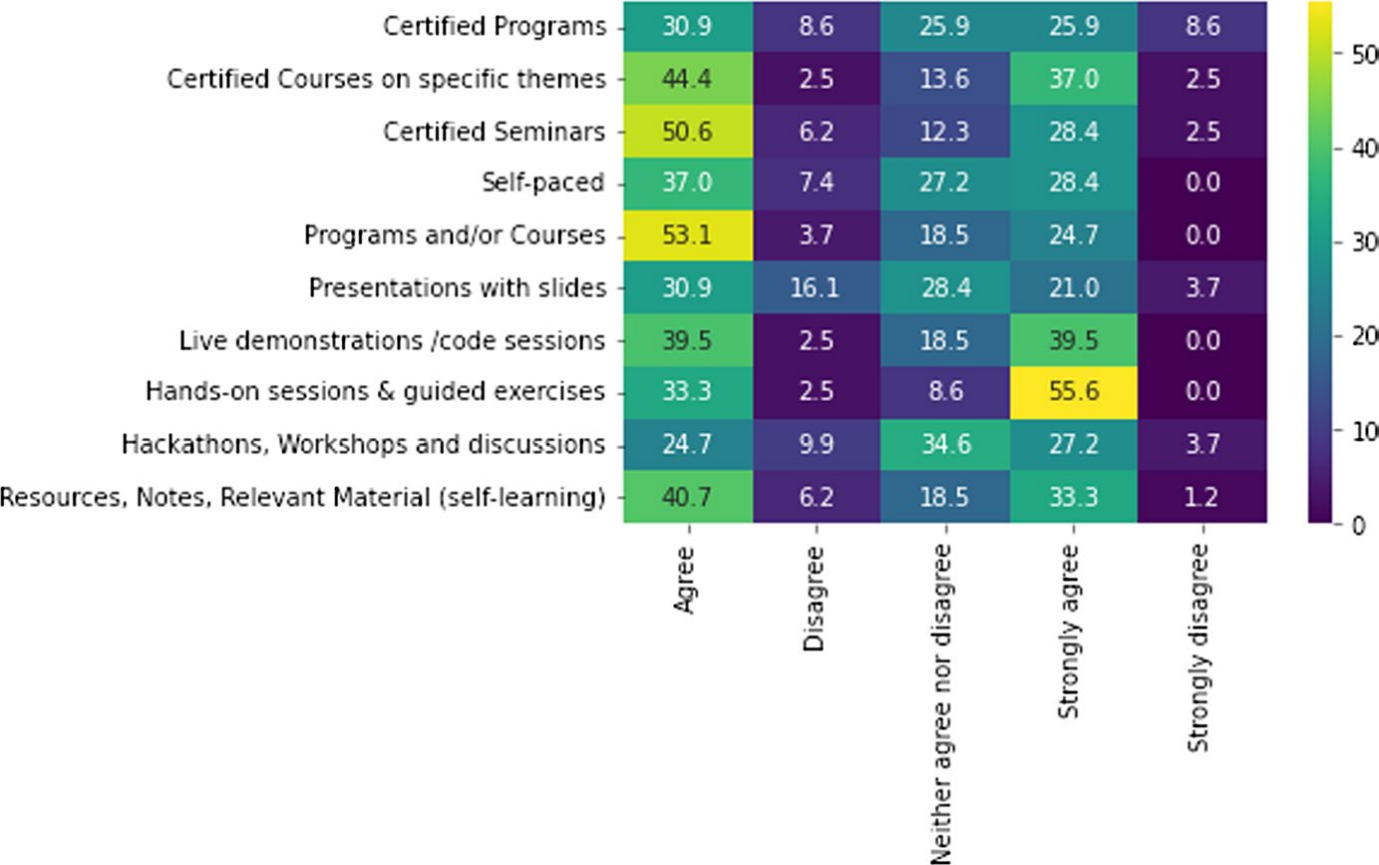
Preferences on type of courses and training [$N=81$]. The *x*-axis is the level of agreement and the *y*-axis the type of training. The coloring goes from dark purple (0%) to yellow (100%) depending on the counts in the sample (color figure online)

**Fig. 6:**
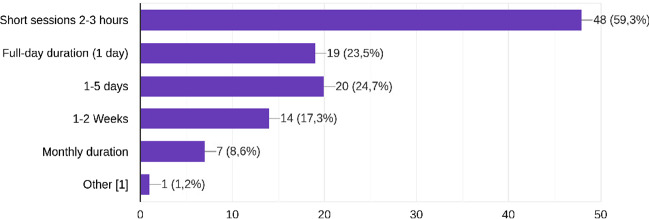
Preferences on duration of courses and training

The last part of the online survey included a user suggestion area for course types and duration that are illustrated and analyzed (see Figs. 5 and 6). In general, the score of all types of courses is high (above 25%). Certification is highly demanded with more than 50% to agree and strongly agree to the provision of certified programs in general (31% agree, strongly agree 26%) (see Fig. 5). More specifically, certified courses on specific themes depict a very high demand, with more than 80% agreeing and strongly agreeing to such courses (44% agree, strongly agree 37%). Finally, the demand for certified seminars in the areas of HPC/HPDA/AI seem to be equally high, with around 79% of respondents agreeing and strongly agreeing to the provision of such seminars (51% agree, 28% strongly agree). Interestingly, “Live demonstration/code sessions” and “Hands-on sessions and guided exercises” are highly preferred forms of training, denoting a horizontal need to provide applied HPC training. In particular, almost 90% prefer hands-on sessions and guided exercises (33% agree and 56% strongly agree) and 80% prefer live demonstration- code sessions (40% agree and 40% strongly agree). Surprising is the fact that hackathons, workshops and discussion appear to be far less preferred among Greek participants with around 52% to prefer such training formats (25% agree and 27% strongly agree). Many reasons may result to that score; (1) the events are too specific with participants from academia or industry having a specific gap in their knowledge that they wish to fill, but those events are not meeting their expectations, or (2) they are too generic trying to present the basic knowledge of HPC to the participants, which are probably already HPC users. Nonetheless, the lack of a HPC training mapping may also lead to those results as it would have provided guidance to the organizers of those events and beyond. Finally, a quite high percentage (74%) of the survey participants seems to prefer the use of self-learning material including HPC/HPDA/AI resources, notes and other relevant material (41% agree and 33% strongly agree). In addition, the “self-paced” choice (60%) scores very high but lower than live demonstration, which is indicative of the complexity and specificity that HPC field presents. A generalized self-paced course may not be suited to include the specific needs of (potential) users of HPC services, compared to a live session where queries and questions will be resolved via the interaction and live conversation.

Independent of the type of training, more than half of the participants of the survey (59.6 %) prefer short sessions 2–3 h. Almost half agree on 1 to some days (23.5% & 24.7%), whereas longer durations present a relatively low demand among the survey respondents. Figure 6 illustrates the duration preferences of survey participants.

## Conclusions

In the current study, the results of the first HPC Training Needs Analysis (TNA) in Greece are presented and analyzed. The scope of this survey study, that was conducted as part of the EuroCC project [22], has been to provide information about the HPC landscape in Greek academia, industry and public sector, and at the same time identify the Greek HPC training needs as those were depicted by the survey respondents during 2021. Due to the relatively low response rate ($N=81$), the study’s findings cannot be generalized [23]. However, it is expected that as the first study of its kind in Greece, the future surveys are expected to have a higher response rate due to higher awareness levels.

Nonetheless, the current online survey revealed that HPC services in Greece are used mostly by academia (e.g., Postgraduate students, University professors). On the other hand, the diffusion of HPC in industry in Greece, appears to be quite low. The most common fields that utilize HPC infrastructures in Greece, based on TNA’s findings, are engineers, natural and computer scientists compared to other fields. The latter may be a result of the sample of participants and/or the kind of research applied to other fields.

**Fig. 7:**
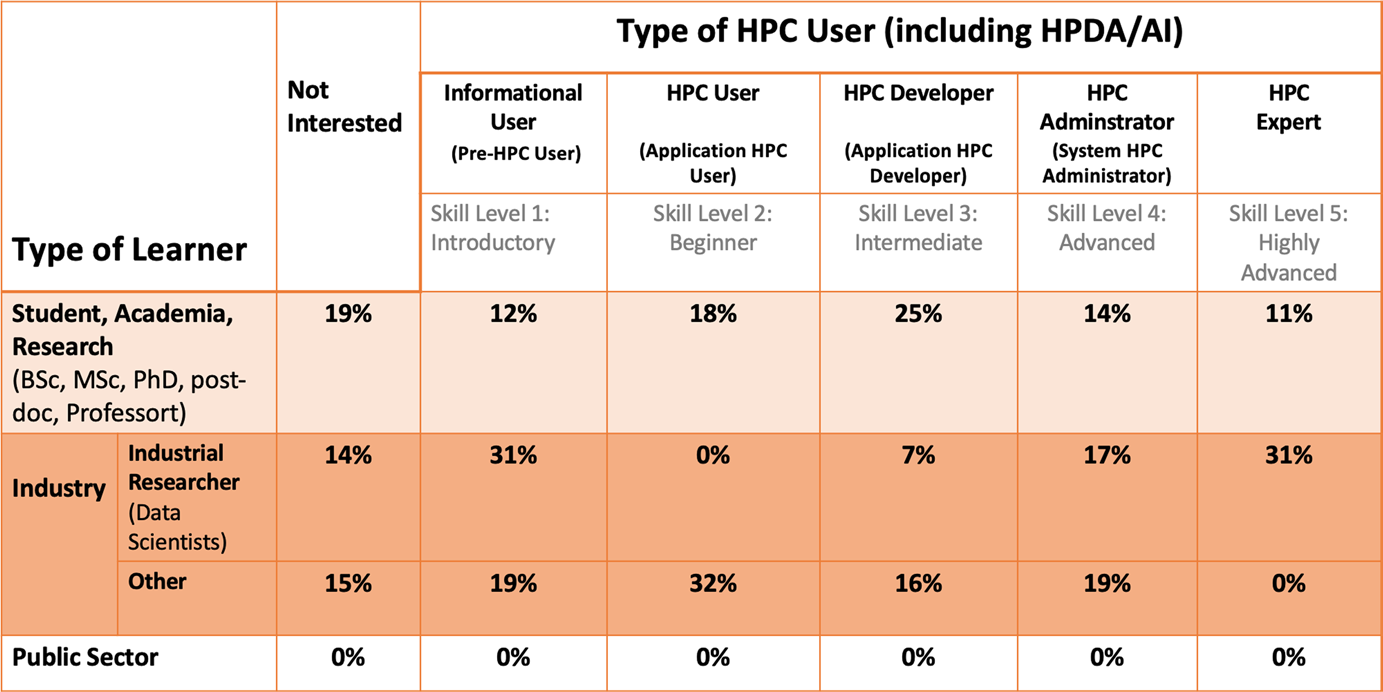
Mapping demand of HPC training in Greece [$N=81$]

The current state of training “demand” for HPC courses in Greece is presented in Fig. 7. The survey confirms the gap in provided HPC training on familiarity level and type, but it is not conclusive for the interest of the industry in HPC services and training as the sample consists mainly of participants working in academia. Nonetheless, the answers of participants derived from the industrial sector indicate that basic training is in demand (as HPC users) and mostly for data analytics and specific programming languages. Overall, it is found that the preferable training scheme is:

1. fast-pace
2. entry level
3. applied HPC training
4. not focused only on HPC
5. provide some kind of certification

As shortcomings we can indicate the small sample size, which is however expected as this is the first study of its kind in Greece. We expect upcoming studies to enrich these findings further and support the broader HPC ecosystem in Greece. The latter may accomplished by performing more targeted campaigns for gathering data, incorporate the questionnaire in the process that academia and industry asking HPC resources and through the collaboration of other relative projects (e.g., EDIH - European Digital Innovation Hub).

As for now, the current study has impacted the next steps of HPC training activities of EuroCC@Greece. As such, trainings targeted to specific groups categorized as less familiar with HPC technology, and material adjusted to this group level of understanding, are being compiled. This will give more tools and opportunities toward a better HPC technology diffusion in academia, industry and public sector in Greece. Under the same scope and taking into consideration the properties of a desired HPC course from the Greek community, an online HPC training for the industrial sector is being created. The latter would be a NOOC (Nano Open Online Course) and it is aspired to be the first step to promote the concept and advantages of HPC usage to the industrial sector of Greece and beyond.

It is noted that the current approach could be relatively easily replicated and used in other countries. Interested parties that wish to undertake the same research for their countries, would undergo only the dissemination and analysis costs/effort providing interesting comparative results.

## Figures and Tables

**Fig. 8:**
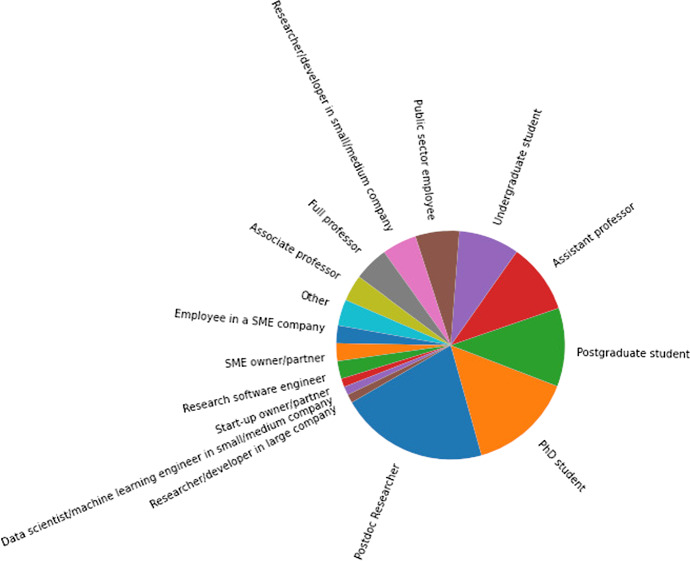
Respondents stakeholder segment [$N=81$] (color figure online)

**Fig. 9:**
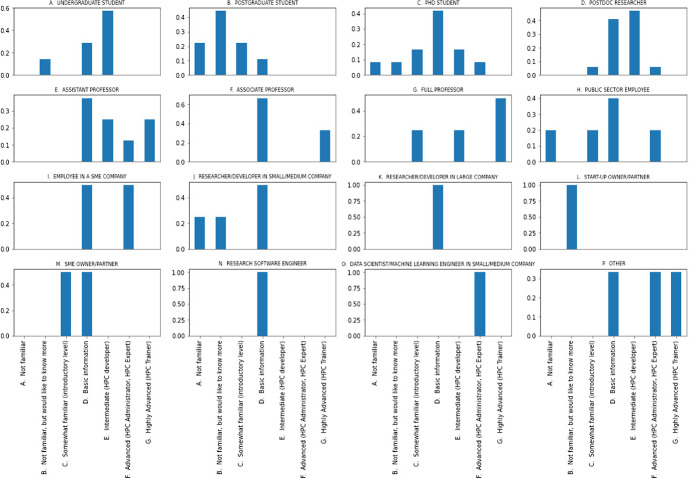
Bar plots familiarity of HPC over type of user [$N=81$]. The individual *x*-axis describes the level familiarity and *y*-axis the percentage of sample per each position

**Fig. 10:**
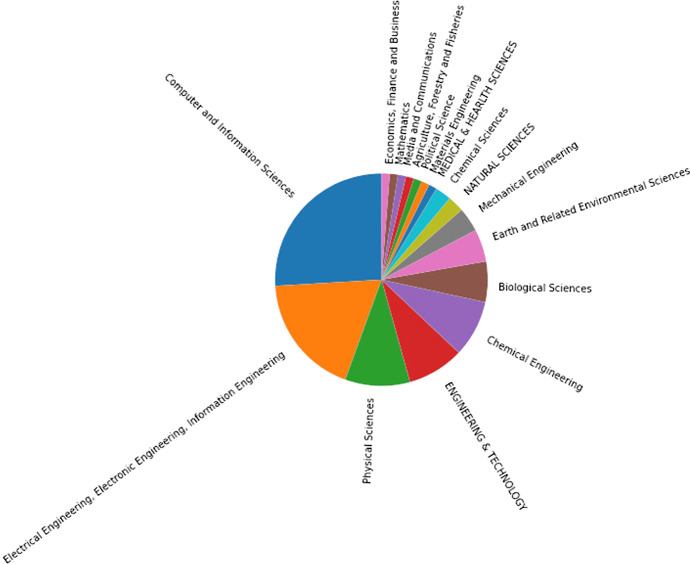
Scientific Domain [$N=81$] (color figure online)

## Data Availability

Survey data analyzed during the current study are available from the corresponding author on reasonable request.

## Appendix A: Supplementary plots

### A.1 User level of education or industry position

Students accounted for 34.5%, postdoctoral researchers for 21% and university professors for 18.5% of the sample. Private sector represented 16% of the respondents whereas the Public sector accounted for 6.2% (Table 1).

**Table 1:** Percentages of user profiles that correspond to pie plot of Fig. 8

Position	Percentage (%)
Postdoc Researcher	21.0
PhD student	14.8
Postgraduate student	11.1
Assistant professor	9.9
Undergraduate student	8.6
Public sector employee	6.2
Researcher/developer in small/medium company	4.9
Full professor	4.9
Associate professor	3.7
Other	3.7
Employee in a SME company	2.5
SME owner/partner	2.5
Research software engineer	2.5
Start-up owner/partner	1.2
Data scientist/machine learning engineer in small/medium company	1.2
Researcher/developer in large company	1.2

Aggregation of the user types (Fig. 3) asked in the survey, was performed according to the following:

- A. Academia/Research: A. Undergraduate student, B. Postgraduate student, C. PhD student, D. Postdoc Researcher, E. Assistant, professor, F. Associate professor G. Full professor
- B. Industry (non-industrial positions): I. Employee in a SME company, J. Researcher/developer in small/medium company, K. Researcher/developer in large company, L. Start-up owner/partner, M. SME owner/partner
- C. S/W Eng, DataScience (Industrial Researchers): N. Research software engineer, O. Data scientist/machine learning engineer in small/medium company

In Fig. 9 the distribution of different type of users are presented (prior to aggregation).

### User scientific background

In terms of scientific domain, an aggregation of different answers to larger groups with common expertise was performed (Table 2). Two new groups were created;

- Natural Sciences: [NATURAL SCIENCES, Physical Sciences, Chemical Sciences, Earth and Related Environmental Sciences, Biological Sciences]
- Engineer & Tech: [ENGINEERING & TECHNOLOGY, Chemical Engineering, Mechanical Engineering, Materials Engineering, Electrical Engineering, Electronic Engineering, Information Engineering]

**Table 2:** Percentages of scientific domain and expertise that correspond to pie plot of Fig. 10

Scientific domain	Percentage
Computer and Information Sciences	25.9%
Electrical Engineering, Electronic Engineering, Information Engineering	18.5%
Physical Sciences	9.9%
ENGINEERING & TECHNOLOGY	8.6%
Chemical Engineering	8.6%
Biological Sciences	6.2%
Earth and Related Environmental Sciences	4.9%
Mechanical Engineering	3.7%
NATURAL SCIENCES	2.5%
Chemical Sciences	2.5%
MEDICAL & HEALTH SCIENCES	1.2%
Materials Engineering	1.2%
Political Science	1.2%
Agriculture, Forestry and Fisheries	1.2%
Media and Communications	1.2%
Mathematics	1.2%
Economics, Finance and Business	1.2%
